# Supplementary material for: African Swine Fever Vaccine Candidate ASFV-G-ΔI177L/ΔLVR Protects Against Homologous Virulent Challenge and Exhibits Long-Term Maintenance of Antibodies
Source: Animals (Basel). 2025 Feb 7;15(4):473. doi: 10.3390/ani15040473 (PMC11851887; doi:10.3390/ani15040473)
Supplement: Supplementary file 1 [file animals-15-00473-s001.zip › animals-3434595-supplementary.pdf]

Table S1: Group A, Detection of vaccine genome copies by qPCR in Rectal and Oral swab samples from the challenge after ASFV-G-ΔI177L/ΔLVR vaccination.

Group			Ct*(qPCR)												
			DPV (Days post-vaccination)									DPC (Days post-challenge)			
			0	4	7	10	14	18	21	25	28	4	7	11	14
$10^{2.25}$ $TCID_{50}$ /dose (IM)	GA1	Rectal	45.00	45.00	45.00	45.00	45.00	45.00	45.00	45.00	45.00	37.9	38.3	37.7	45.00
		Oral	45.00	45.00	45.00	37.3	45.00	45.00	45.00	45.00	45.00	37.4	38.8	45.00	35.8
	GA2	Rectal	45.00	45.00	45.00	45.00	45.00	45.00	45.00	45.00	45.00	36.2	45.00	32.1	34.6
		Oral	45.00	45.00	45.00	45.00	45.00	45.00	45.00	45.00	45.00	37.2	36.0	32.2	33.4
	GA3	Rectal	45.00	45.00	45.00	36.8	45.00	45.00	28.5	31.8	38.5	45.00	36.4	31.4	37.9
		Oral	45.00	45.00	38.2	37.5	45.00	37.7	38.3	37.7	36.7	38.4	34.6	30.8	32.4
	GA4	Rectal	45.00	45.00	45.00	37.9	45.00	38.7	31.5	45.00	45.00	45.00	38.2	38.6	26.8
		Oral	45.00	45.00	45.00	45.00	45.00	38.7	45.00	39.6	45.00	37.3	35.9	45.00	34.2
Challeng e Control	GA-1-1	Rectal	45.00	45.00	45.00	45.00	45.00	45.00	45.00	45.00	45.00	38.3	22.9	D	D
		Oral	45.00	45.00	45.00	45.00	45.00	45.00	45.00	45.00	45.00	38.3	22.9	D	D
	GA-1-2	Rectal	45.00	45.00	45.00	45.00	45.00	45.00	45.00	45.00	45.00	37.0	27.9	D	D
		Oral	45.00	45.00	45.00	45.00	45.00	45.00	45.00	45.00	45.00	37.0	27.9	D	D
	GA-1-3	Rectal	45.00	45.00	45.00	45.00	45.00	45.00	45.00	45.00	45.00	37.4	29.9	D	D
		Oral	45.00	45.00	45.00	45.00	45.00	45.00	45.00	45.00	45.00	38.4	27.9	D	D
	GA-1-4	Rectal	45.00	45.00	45.00	45.00	45.00	45.00	45.00	45.00	45.00	37.6	24.9	D	D
		Oral	45.00	45.00	45.00	45.00	45.00	45.00	45.00	45.00	45.00	36.9	27.7	D	D
Negative Control	GA-2-1	Rectal	45.00	45.00	45.00	45.00	45.00	45.00	45.00	45.00	45.00	45.00	45.00	45.00	45.00
		Oral	45.00	45.00	45.00	45.00	45.00	45.00	45.00	45.00	45.00	45.00	45.00	45.00	45.00
	GA-2-2	Rectal	45.00	45.00	45.00	45.00	45.00	45.00	45.00	45.00	45.00	45.00	45.00	45.00	45.00
		Oral	45.00	45.00	45.00	45.00	45.00	45.00	45.00	45.00	45.00	45.00	45.00	45.00	45.00
	GA-2-3	Rectal	45.00	45.00	45.00	45.00	45.00	45.00	45.00	45.00	45.00	45.00	45.00	45.00	45.00
		Oral	45.00	45.00	45.00	45.00	45.00	45.00	45.00	45.00	45.00	45.00	45.00	45.00	45.00
	GA-2-4	Rectal	45.00	45.00	45.00	45.00	45.00	45.00	45.00	45.00	45.00	45.00	45.00	45.00	45.00
		Oral	45.00	45.00	45.00	45.00	45.00	45.00	45.00	45.00	45.00	45.00	45.00	45.00	45.00

*Ct<45 was considered positive. **D:death

Table S2: Group B (Long-term), Vaccine genome copy using qPCR in Rectal and Oral swab samples after ASFV-G-ΔI177L/ΔLVR vaccination.

**Group**			**Ct*(qPCR)**																
			**DPV (Days post-vaccination)**																
			**0**	**4**	**7**	**11**	**14**	**18**	**21**	**24**	**28**	**32**	**35**	**39**	**42**	**46**	**49**	**53**	**56**
10^3 $TCID_{50}$ /dose (IM)	GB1	Rectal	45.00	45.00	39.9	45.00	36.5	40.0	45.00	35.1	45.00	45.00	45.00	40.3	45.00	45.00	45.00	45.00	45.00
		Oral	45.00	45.00	45.00	39.7	39.9	38.4	45.00	45.00	40.6	45.00	45.00	45.00	45.00	45.00	39.2	45.00	40.4
	GB2	Rectal	45.00	45.00	40.0	40.5	39.8	38.6	45.00	39.7	45.00	45.00	40.2	37.3	45.00	45.00	45.00	45.00	45.00
		Oral	45.00	45.00	39.2	39.5	39.5	45.00	39.2	45.00	45.00	45.00	39.0	45.00	39.7	45.00	40.5	45.00	45.00
	GB3	Rectal	45.00	45.00	45.00	45.00	45.00	40.6	45.00	45.00	45.00	38.5	45.00	45.00	45.00	45.00	45.00	45.00	45.00
		Oral	45.00	45.00	45.00	45.00	45.00	45.00	45.00	45.00	45.00	39.1	45.00	38.8	45.00	45.00	38.3	36.5	40.4
	GB4	Rectal	45.00	45.00	45.00	45.00	45.00	45.00	45.00	45.00	38.3	38.0	45.00	39.2	38.2	39.0	39.0	45.00	45.00
		Oral	45.00	45.00	45.00	45.00	40.8	45.00	40.6	39.6	37.8	45.00	45.00	39.1	45.00	45.00	45.00	36.6	45.00
Negative Control	GB-1-1	Rectal	45.00	45.00	45.00	45.00	45.00	45.00	45.00	45.00	45.00	45.00	45.00	45.00	45.00	45.00	45.00	45.00	45.00
		Oral	45.00	45.00	45.00	45.00	45.00	45.00	45.00	45.00	45.00	45.00	45.00	45.00	45.00	45.00	45.00	45.00	45.00
	GB-1-2	Rectal	45.00	45.00	45.00	45.00	45.00	45.00	45.00	45.00	45.00	45.00	45.00	45.00	45.00	45.00	45.00	45.00	45.00
		Oral	45.00	45.00	45.00	45.00	45.00	45.00	45.00	45.00	45.00	45.00	45.00	45.00	45.00	45.00	45.00	45.00	45.00

*Ct<45 was considered positive.
